# Vestibular dysfunction in patients with obstructive sleep apnea: from physiopathology to treatment

**DOI:** 10.3389/frsle.2026.1750104

**Published:** 2026-10-09

**Authors:** Marrakchi Jihène, Jouini Selima, Sifaoui Aymen, Driss Melek

**Affiliations:** ENT Department of Menzel Bourguiba Hospital, Bizerte, Tunisia

**Keywords:** apnea, balance, disorders, obstructive, sleep, vertigo

## Abstract

Sleep disorders include a wide variety of conditions that can have serious consequences on general health. The most common sleep problem related to breathing is obstructive sleep apnea (OSA). The Ear, Nose, and Throat (ENT) manifestations of this pathology are numerous including balance disorders. Understanding the impact of OSA on the vestibular function in Meniere's disease, vestibular migraine, and benign paroxysmal positional vertigo ensure a systematic screening and an appropriate care.

## 1 Introduction

Sleep disorders include a wide variety of conditions that can have serious consequences on general health. The most common sleep problem related to breathing is obstructive sleep apnea (OSA). Nowadays, it represents a real health problem (Lyons et al., 2020) as it has various clinical impacts. Many studies seek to identify the various repercussions of this pathology on multiple systems of human body with the aim of treating these impacts and improving the quality of life. TheEar, Nose and Throat (ENT) manifestations of this pathology are numerous and include the following: xerostomia (Pico-Orozco et al., 2020), Eustachian tube dysfunction (Choudhury and Deshmukh, 2023), nocturnal stridor (Dedhia et al., 2014), and balance disorders (Manuele and Antonio, 2023). In the last decade, several authors have focused attention on research to verify the functional neurotological alterations in OSA in particularly on the vestibular system (Gallina et al., 2010). The various people involved in the diagnosis, treatment, and follow-up of OSA must be aware of the risk of developing ENT disorders related to this pathology.

Sleep apnea syndrome (SAS) is defined as the occurrence of apneas and/or hypopneas during sleep. These respiratory events are defined as the interruption (apnea) or reduction (hypopnea) of more than 30% of the inspiratory flow, lasting more than 10 seconds and occurring more than 5 times per hour of sleep. Obstructive apneas are distinguished from central apneas according to whether the respiratory events are linked to an obstruction of the upper airways or to a respiratory control disorder. Obstructive Sleep Apnea Syndrome (OSAS) is characterized by respiratory events (apneas or hypopneas) associated with clinical symptoms, such as excessive daytime sleepiness, or documented cardiovascular comorbidities.

The aim of this article is to compile and therefore understand the impact of OSA on the vestibular function in Meniere's disease, vestibular migraine, and benign paroxysmal positional vertigoto ensure asystematicscreening and an appropriate care.

## 2 Methodology

This literature review was conducted to evaluate the current understanding of the relationship between OSA and vestibular dysfunction. A systematic search was performed across the PubMed and ScienceDirect databases to identify relevant peer-reviewed literature published between 2010 and 2024.

The search strategy utilized combinations of the following keywords: “Obstructive Sleep Apnea,” “OSA,” “vestibular dysfunction,” “vertigo,” “Meniere's disease,” “Benign Paroxysmal Positional Vertigo (BPPV),” and “vestibular migraine.”

To ensure the rigor and relevance of the analyzed data, the following criteria were applied:

**Inclusion criteria:**

- Study Type: Peer-reviewed original research, clinical trials, and observational studies.
- Population: Adult patients (aged 18 and older) diagnosed with OSA.
- Diagnostic Threshold: Patients must have an Apnea-Hypopnea Index (AHI) ≥ 5, confirmed via polysomnography or polygraphy.
- Outcome Measures: Studies assessing vestibular function using objective tests [e.g., vHIT (video head impulse test), VEMP (vestibular-evoked myogenic potentials), VOR gain (vestibulo-ocular reflex)], or validated subjective scales (e.g., Dizziness Handicap Inventory).

**Exclusion criteria:**

- Study Design: Case reports, editorials, and conference abstracts were excluded to maintain a high level of evidence.
- Confounding Factors: Studies involving patients with pre-existing central neurological disorders unrelated to OSA or those with incomplete diagnostic sleep data.
- Language: Non-English or non-French publications (depending on your specific scope).

A total of 31 articles met these criteria and were included in this review for final analysis.

## 3 Results

### 3.1 Obstructive sleep apnea and vestibular dysfunction

OSA can occur in any age group, but prevalence increases between middle and older age. About 80 percent to 90 percent of adults with OSA remain undiagnosed (Gottlieb and Punjabi, 2020). Estimated current United States prevalence at 32.4% (83.7 million people), showing a massive increase from 1990s data (Sönmez et al., 2025).

Diagnosis requires the presence of clinical criteria A or B and polysomnographic criteria C:

A: excessive daytime sleepiness not explained by other factors.

B: At least two of the following criteria not explained by other factors [severe and daily snoring, sensations of choking or suffocation during sleep, non-restorative sleep, daytime fatigue, difficulty concentrating, and nocturia (more than one micturition per night)].

C: polysomnographic or polygraphic criteria: apneas + hypopneas ≥ 5 per hour of sleep (apnea hypopnea index [AHI] ≥ 5) (American Academy of Sleep Medicine, 2014).

The severity will be judged on the frequency of occurrence of abnormal respiratory events: mild: between 5 and 15 events per hour; moderate: between 15 and 30 events per hour and severe: 30 or more events per hour.

The pathogenesis of OSA involves a complex interplay between anatomical predisposition and non-anatomical ‘PALM' endotypes (P-crit, Arousal threshold, Loop gain, and Muscle responsiveness) (Kang et al., 2025). While sleep-induced hypotonia of the genioglossus and upper airway dilator muscles is central, the collapse is fundamentally dictated by the transmural pressure exceeding the pharyngeal critical closing pressure. In susceptible individuals, the loss of wakefulness-related drive, coupled with inadequate compensatory neuromuscular recruitment, leads to recurrent pharyngeal collapse, intermittent nocturnal hypoxemia, and significant intrathoracic pressure swings (Alessandrini et al., 2019).

Most people with OSA snore loudly and frequently, with periods of silence when airflow is reduced or blocked. Then, they make choking, snorting, or gasping sounds when their airway reopens (Gottlieb and Punjabi, 2020).

Diagnosis requires the presence of clinical criteria and polysomnographic criteria C.

The severity will be judged on the frequency of occurrence of abnormal respiratory events: mild: between 5 and 15 events per hour; moderate: between 15 and 30 events per hour and severe: 30 or more events per hour.

#### 3.1.1 What constitutes vestibular dysfunction?

Vestibular dysfunction can be defined as:

- Objective deficits: A reduction in VOR gain (typically on Video Head Impulse Test/vHIT), presence of abnormal saccades, or reduced caloric response (unilateral weakness).
- Physiological markers: Asymmetrical or absent signals in VEMPs (Vestibular Evoked Myogenic Potentials), indicating damage to the otolith organs (saccule/utricle).
- Functional impairment: An inability of the vestibular system to maintain spatial orientation and gaze stability during head movement, often manifesting as vertigo, oscillopsia, or imbalance.

The symptoms of vestibular dysfunction in patients with OSA can vary widely and may include dizziness, vertigo, imbalance, spatial disorientation, and problems with coordination. These symptoms can significantly impact daily activities, quality of life, and increase the risk of falls and accidents (Stevens et al., 2020).

### 3.2 Impact of OSA on vestibular function

#### 3.2.1 General findings

A study conducted by Micarelli which aimed to explore the vestibular impairment in moderate-to- severe obstructive sleep apnea all the patients with OSA (32 patients) showed a decrease of VOR gain VHIT compared to healthy subjects (Micarelli et al., 2017). This suggests a vestibular disorder in OSA syndrome patients.

The OSA syndrome patients showed highest Dizziness Handicap Inventory scores and the lowest VOR gain, but statistical significance was not found (Bae et al., 2022).

The VEMPs: ocular VEMP (oVEMP) and cervical VEMP (cVEMP) appear to be significantly lower in OSA syndrome patients than those in the control group (Xu et al., 2022).

The moderate-severe OSA syndrome group had a significantly higher fall risk index compared to both the mild OSA syndrome group and the control group (Soyaliç et al., 2017).

Recent meta-analyses have quantified the risk of vestibular damage in OSA patients. A study from February 2025 indicates that adults with OSA are significantly more likely to have abnormal results in vestibular testing, with an odds ratio (OR) of 4.8 for abnormal caloric tests, 7.9 for absent cVEMP, and 6.2 for absent oVEMP (Kang et al., 2025). This suggests that OSA induces structural and functional impairments across the semicircular canals, the saccule, and the utricle.

Furthermore, research published in March 2025 found a staggering 75% prevalence of OSA in patients diagnosed with Meniere's Disease, with those in the moderate-to-severe OSA category experiencing more significant hearing loss (Petry et al., 2025).

#### 3.2.2 Physiopathology

Various theories have been put forward to explain the relationship between OSA and vestibular dysfunction.

##### 3.2.2.1 The ischemic theory

Dizziness is frequently reported by the patient with OSA (Tsai et al., 2018). Several studies show that OSA is an independent risk factor for hypertension, myocardial infarction, ischemic attacks and stroke. This results from intermittent hypoxia, increased sympathetic tone, impaired endothelial functions, and various neurohumoral factors (Bouzerda, 2018). The reasons for this sympathetic hypertonia remain unclear, although hyperstimulation of chemoreceptors may be possible (Narkiewicz et al., 1999). In the absence of overt cardiac or vascular pathology, patients suffering from OSA have endothelial dysfunction (Kato et al., 2000). The hypoxemia, hypercapnia, and variations in blood pressure that accompany episodes of obstructive apnea are powerful stimuli for the release of vasoactive substances and alter the function of the endothelium. Elevated levels of endothelin, probably in response to the hypoxemic phases of OSA, contribute to sustained vasoconstriction and various cardiovascular disorders (Phillips et al., 1999). Due to the rapid changes of blood flow, it is difficult for the cerebral autoregulation system to avoid cerebral ischemia (Byun et al., 2022). As the inner ear receives all its arterial inflow viaterminal branches of the anterior inferior cerebellar artery without collateral circulations, vasospasm, andocclusion of vessels in the head could result in limited flow to the inner ears. Also, sleep deprivation as a result of OSA can lead to a lack of postural stability and adaptability (Gomez et al., 2008). The reduced blood flow may have a detrimental effect on the health and function of the vestibular organs, leading to dysfunction. Another potential mechanism is related to the physiological changes that occur due to OSA. The repeated episodes of apnea and subsequent arousal from sleep can result in autonomic nervous system dysfunction. This dysfunction may disrupt the normal control and regulation of blood flow to the inner ear, leading to vestibular dysfunction (Kim et al., 2022).

Laboratory studies have shown that peripheral vestibular organs and the vestibular nucleus are willing to be damaged by hypoxia or reactive oxygen species (Ciorba et al., 2010; Lee et al., 2017).

##### 3.2.2.2 The inflammation theory

Furthermore, the chronic inflammation resulting from OSA may also contribute to vestibular dysfunction. Inflammation plays a significant role in the pathophysiology of OSA, and it has been suggested that the inflammatory mediators released during the inflammatory process could have detrimental effects on the vestibular system (Liu et al., 2020). In fact, chronic inflammation triggers pro-inflammatory cytokines (TNF-α, IL-6) which induce apoptosis in vestibular hair cells and cause atrophy of the stria vascularis (Di Stadio et al., 2025). Besides, Inflammation increases monocyte activation, leading to microvascular perfusion deficits within the inner ear, potentially causing localized ischemia of the vestibular neuroepithelium (Sönmez et al., 2025).

##### 3.2.2.3 The anatomical theory

An anatomical explanation has been put forward by certain authors. In fact, the nasopharynx and oropharynxwhich are important in maintaining the physiological function of the eustachian tube, are the sites in the airway that collapses the mostin OSA. The eustachian tube connects the nasopharynx to the middle ear. Any changes in the function of the nasopharynx affects the middle ear pression which can damage the activity of the vestibular neurons and lead to vestibular dysfunction (Cheung et al., 2022). The repetitive mechanical stress and high negative intrathoracic pressure during obstructive events can lead to secondary Eustachian tube dysfunction. This dysfunction alters middle ear pressure, which may indirectly affect the inner ear fluids (endolymph and perilymph) and stimulate the vestibular receptors, potentially triggering vertiginous symptoms or postural instability.

Gallina et al. (2010) suggest that the functional alteration of the vestibular nuclei may be an indication of abnormal activity of the respiratory nuclei considering the anatomical contiguity of this center.

Beyond the ischemic and inflammatory theories, recent evidence highlights autonomic dysregulation. A 2025 study demonstrated a direct correlation between semicircular canal dysfunction and altered heart rate variability (HRV) in OSA patients, suggesting that the autonomic nervous system is a critical mediator in the “vertigo-apnea” (Wang et al., 2025).

### 3.3 Benign paroxysmal positional vertigo

BPPVis the most common cause of vertigo worldwide (Kim et al., 2021). It occurs when small calcium crystals in the inner ear, called otoconia, become dislodged and migrate into one of the fluid-filled canals responsible for detecting head movements. These misplaced crystals can then interfere with the normal fluid flow in the canal, causing a false sense of movement (Cole and Honaker, 2022).

The characteristic symptom of BPPV is sudden episodes of intense vertigo that last for a short duration, typically a few seconds up to a minute. These episodes are often triggered by specific head movements, such as turning in bed, tilting the head, or looking up or down. Additional symptoms may include dizziness, unsteadiness, nausea, and visual disturbances (Xiang-Dong, 2011). A 2021 study, specifically investigated whether Obstructive Sleep Apnea acts as a precursor to peripheral vestibular disorders like BPPV. It concluded that BPPV is not just a random occurrence in these patients but a systemic consequence of sleep-disordered breathing (Byun et al., 2022).

BPPV is typically diagnosed through a physical examination and is confirmed with the help of specialized tests, such as the Dix-Hallpike or Roll maneuvers (Cole and Honaker, 2022).

It seems to be little literature on the relation between BPPV and OSA and to the best of our knowledge, no study has ever been carried out to look specifically for a BPPV in patients with OSA. In a research conducted by Xue to investigate the differences of sleep disorders between vestibular migraine and benign paroxysmal positional vertigo, the proportion of patients with OSA was significantly higher in the BPPV group than in the healthy controls group (Xue et al., 2021).

### 3.4 Meniere's disease

When Meniere's disease and OSA occur together, the combination can have a profound negative effect on the affected individual's overall health and wellbeing. About 40% of Meniere's disease patients suffer from inadequate sleep (Takahashi et al., 2005). It is reported that OSA is seen in approximately 15% of Meniere's disease (Nakayama and Kabaya, 2013). In a nationwide 9-year longitudinal cohort database of 1,025,340 South Korean patients, patients aged between 45 and 64 years with OSA were independently associated with a 2-fold higher incidence of subsequent Meniere's disease compared to those without OSA (Kim et al., 2019).

Meniere's disease is a chronic disorder of the inner ear that affects balance. It is characterized by various symptoms such as vertigo, hearing loss, tinnitus and a feeling of fullness or pressure in the affected ear. Meniere's disease can have a significant impact on an individual's quality of life, as it can disrupt daily activities and cause unpredictable episodes of dizziness and imbalance. These episodes may last for a few minutes to several hours, leaving individuals feeling exhausted.

The physiopathology of this disease hasn't been completely elucidated. It is thought to occur due to the accumulation of endolymphatic fluid in the cochlea and the vestibular organ (idiopathic endolymphatic hydrops) (Koenen and Andaloro, 2019). Some studies suggest that vascular occlusion may contribute to Meniere's disease (Gortan, 2000; Nakai et al., 1992).

The relationship between Meniere's disease and OSA is not yet fully understood. One possible explanation is the shared risk factors between the two conditions. Both Meniere's disease and OSA have been associated with obesity, hypertension, and metabolic syndromes, which could contribute to the development of both disorders (Kim et al., 2019). Additionally, the intermittent hypoxia experienced in OSA may affect the inner ear's oxygen supply, potentially worsening the vertigo and tinnitus in Meniere's disease. During episodes of apnea in OSA patients, there is a decrease in oxygen which leads to oxygen deprivation in various organs, including the inner ear. This can potentially exacerbate the underlying pathology of Meniere's disease. In fact, the blood labyrinthine barrier is critical in the maintenance of inner-ear homeostasis and the intermittent hypoxic events in OSA increase oxidative stress (Low and Lim, 2021). This is consistent with the ischemic theory explaining vestibular dysfunction in OSA.

Moreover, the mechanisms behind both Meniere's disease and OSA share some similarities. Both conditions have been associated with inflammation, immune system dysfunction, and impaired fluid regulation in the body. These underlying factors may contribute to the development and progression of both disorders (Liu et al., 2020; Frejo and Lopez-Escamez, 2022).

Furthermore, sleep disturbances resulting from OSA can exacerbate the symptoms of Meniere's disease. Patients with OSA frequently experience fragmented sleep patterns due to repeated awakenings from apnea episodes. These disrupted sleep patterns can have a negative impact on the quality of life and may contribute to worsening symptoms experienced by Meniere's disease patients specially tinnitus (Asnis et al., 2018).

### 3.5 Vestibular migraine

Vestibular migraine is the most common cause of episodic (non-positional) vertigo in adults as well as in children (Stolte et al., 2015). It is characterized by episodes of vertigo or dizziness, often accompanied by other migraine symptoms such as headache, nausea, and sensitivity to light and sound. These episodes typically last for several minutes to hours and can be disabling for the individual. Unlike typical migraines, vestibular migraines do not always cause a headache (Stolte et al., 2015). The Bárány Society, which represents the international community of basic scientists, otolaryngologists, and neurologists committed to vestibular research, developed diagnostic criteria for vestibular migraine (Dieterich et al., 2016).

The exact cause of vestibular migraines is unknown, although several hypotheses have been proposed to explain the symptoms: activation of the trigemino-vascular system, release of pro-inflammatory mediators and vasodilatation (Espinosa-Sanchez and Lopez-Escamez, 2015). Obesity, which is a risk factor for OSA, is also a predisposing factor for migraine (Tiseo et al., 2020).

There has been little investigation into vestibular migraine in patients with OSA. The prevalence of vestibular migraine in the OSA patients was 10.8% (on 130 patients with severe OSA) in a study conducted by Tungvachirakul (2022). In a research conducted by Xue, the proportion of patients with OSA was significantly higher in the vestibular migraine group than in the healthy controls group (Xue et al., 2021).

It is important to recognize OSA as a trigger of vestibular migraine. In fact, treatment of OSA could help solving vestibular migraine and should be investigated further in the future (Tungvachirakul, 2022).

### 3.6 Therapeutic implications and potential benefits of OSA treatment on vestibular function

Treatment methods for OSA include besides behavioral modification, continuous positive airway pressure (CPAP), mandibular advancement devices, and surgical treatment. CPAP remains the gold standard (Arredondo et al., 2022). To our knowledge, few studies have assessed the impact of CPAP on vestibular function, and none have evaluated the effect of surgery.

OSA treatment, through CPAP therapy, has shown promising effects on improving vestibular function. CPAP therapy involves the use of a mask to deliver a constant flow of air pressure, preventing the airway from collapsing during sleep. By effectively treating OSA, CPAP therapy has demonstrated positive outcomes in patients' overall wellbeing, including a potential improvement in vestibular function (Alessandrini et al., 2019). Alessandrini et al. (2019) compared VOR gains by performing VHIT before and after 12 months of CPAP treatment in patients with moderate-to-severe OSA. VOR gain did not demonstrate significant improvement, but it might provide further perspectives for future studies to assess VOR gain changes after longer periods of CPAP treatment (Alessandrini et al., 2019).

By reducing the frequency of breathing interruptions during sleep, CPAP therapy helps to maintain consistent oxygen levels and minimizes oxidative stress, which may positively impact the vestibular system. Furthermore, it has been observed that treating OSA can lead to a decrease in inflammation levels, which could be beneficial for vestibular function (Nakayama et al., 2015).

The association between Meniere's disease and OSA underscores the importance of comprehensive evaluation and management in patients presenting with either condition. It is crucial for healthcare professionals to recognize the potential coexistence of these two disorders to provide appropriate treatment. Continuous positive airway pressure (CPAP) therapy, which is the primary treatment for OSA, may also benefit Meniere's disease patients by improving sleep quality and reducing the frequency and severity of Meniere's disease symptoms (Nakayama et al., 2015). Treatment options for Meniere's disease frequently include drugs that have hypnotic, muscle relaxing, and antianxiety properties (Takeno et al., 2006; Brookes, 1996). Those medications can worsen OSA symptoms (Nakayama and Kabaya, 2013). Managing the symptoms and consequences of both disorders requires a comprehensive approach, involving lifestyle modifications, pharmacological interventions, and potentially surgical procedures. Nakayama suggested in a study conducted over 20 patients that solitary CPAP therapy may become a new effective treatment strategy for Meniere's disease patients with OSA, for control of dizziness and vertigo and potential benefit of hearing (Nakayama et al., 2015).

Treatment of OSA syndrome with continuous positive airway pressure is associated with improvements in sleep quality and migraine in terms of reducing the average attack frequency per month (from 5.8 to 0.1 days), average duration of attacks, pain intensity, average number of pain attacks of attacks days of inability to work, and acute drug use (Tiseo et al., 2020).

## 4 Conclusion

This review highlights the significant interplay between Obstructive Sleep Apnea (OSA) and the vestibular system. The evidence suggests that intermittent hypoxia, chronic inflammation, and vascular compromise serve as the primary pathophysiological links leading to impaired vestibulo-ocular reflex (VOR) and increased fall risks.

Integrating systematic vestibular screening into the clinical management of OSA patients—particularly those suffering from Meniere's disease or BPPV—is essential. Conversely, patients presenting with unexplained balance disorders should be evaluated for underlying sleep-disordered breathing. Future research should focus on the impact of CPAP therapy on reversing these vestibular deficits to optimize patient quality of life and safety.
